# Early serum creatinine accurately predicts acute kidney injury post cardiac surgery

**DOI:** 10.1186/s12882-017-0504-y

**Published:** 2017-03-16

**Authors:** Keren Grynberg, Kevan R. Polkinghorne, Sharon Ford, Fiona Stenning, Thomas E. Lew, Jonathan A. Barrett, Shaun A. Summers

**Affiliations:** 1 Department of Nephrology, Monash Medical Centre, Level 3, Block E, 246 Clayton Rd, Clayton, VIC 3168 Australia; 2 Centre for Inflammatory Diseases, Department of Medicince, Monash University, Monash Medical Centre, 246 Clayton Rd, Clayton, VIC 3168 Australia; 3 Department of Epidemiology and Preventative Medicine, Monash University, Commercial Road, Prahran, VIC 3181 Australia; 4 Department of Medicine, Monash University, Monash Medical Centre, Level 5, Block E, 246 Clayton Rd, Clayton, VIC 3168 Australia; 5 Intensive Care Unit, Cabrini Hospital, Wattletree Road, Malvern, VIC Australia

**Keywords:** Acute Kidney Injury, AKIN criteria, Biomarkers, Cardiac surgery, Serum creatinine

## Abstract

**Background:**

Acute Kidney Injury (AKI) is a well recognized complication of cardiac surgery. It is associated with significant morbidity and mortality. The aims of our study are twofold;

1. To define the incidence of AKI post cardiac surgery.
2. To identify pre-morbid and operative risk factors for developing AKI and to determine if immediate post operative serum creatinine (IPOsCr) accurately predicts the development of AKI.

**Methods:**

We prospectively studied 196 consecutive patients undergoing elective (on-pump) cardiac surgery. Baseline patient characteristics, including medical co-morbidities, proteinuria, procedural data and kidney function (serum creatinine (sCr) were collected. Internationally standardised criteria for AKI were used (sCr >1.5 times baseline, elevation in sCr >26.4 μmmol/L (0.3 mg/dl). Measurements were collected pre-operatively, within 2 h of surgical completion (IPOsCr) and daily for two days. Logistic regression was used to assess predictive factors for AKI including IPOsCr. Model discrimination was assessed using ROC AUC curves.

**Results:**

Forty (20.4%) patients developed AKI postoperatively. Hypertension (OR 2.64, *p* = 0.02), diabetes (OR 2.25, *p* = 0.04), proteinuria (OR 2.48, *p* = 0.02) and a lower baseline eGFR (OR 0.74, *p* = 0.002) were associated with AKI in univariate analysis. A multivariate logistic model with preoperative and surgical factors (age, gender, eGFR, proteinuria, hypertension, diabetes and type of cardiac surgery) demonstrated moderate discrimination for AKI (ROC AUC 0.76). The addition of IPOsCr improved model discrimination for AKI (AUC 0.82, *p* = 0.07 versus baseline AUC) and was independently associated with AKI (OR 7.17; 95% CI 1.27–40.32; *p* = 0.025).

**Conclusions:**

One in 5 patients developed AKI post cardiac surgery. These patients have significantly increased morbidity and mortality. IPOsCr is significantly associated with the development of AKI, providing a cheap readily available prognostic marker.

## Background

Acute Kidney Injury (AKI) is a common and serious complication of cardiac surgery. Mild AKI occurs in nearly one in five patients undergoing cardiac surgery and is associated with a 19-fold increase in short-term mortality [1]. Severe renal injury, requiring replacement therapy, occurs in 2% of patients; in these cases mortality rates approach 60% [2]. In addition to heightened morbidity and mortality, patients who develop AKI have longer hospital admissions, which increase the financial burden on healthcare systems [1]. AKI is also a risk factor for the development of chronic kidney disease (CKD) and its many sequelae [3].

Pre and peri-operative risk factors, including gender, advancing age, medical co-morbidities, complex surgery and extensive use of inotropes have been shown to increase the risk of AKI [1, 4]. While this allows for risk stratification, it does not help to identify individual patients who will develop AKI in the post-operative period. Historically and again recently, it has been shown that early nephrology referral for patients who develop AKI results in improved outcomes [5–7]. Hence it is important to identify patients who are at risk of developing AKI post cardiac surgery.

Two strategies have been used in an attempt to improve the ability to diagnose AKI in a timely manner. Firstly, novel biomarkers, including urine interleukin (IL)-18 and serum neutrophil gelatinase-associated lipocalin (NGAL), have been shown to increase diagnostic accuracy [8] and to predict outcomes [9]. Currently these biomarkers are expensive and are not routinely available. Alternatively, the use of conventional tests, which are more readily available, has also shown promise. Ho et al. showed that measurement of serum creatinine, within 6 h of completion of cardiac surgery, significantly improved the prediction of AKI [10]. This is an important observation and could be widely applicable as creatinine is cheap and readily available, even in resource poor settings.

In this study we aimed to define the incidence of AKI post cardiac surgery, to identify pre-morbid and operative risk factors for developing AKI and to accurately predict patients at risk of AKI using routine biochemical tests. We hypothesized that an early serum creatinine, performed within 2 hours of the cessation of surgery would be predictive of AKI, allowing early identification of at risk patients.

## Methods

### Setting and study population

This is a single center, prospective observational cohort study of all subjects undergoing elective, on pump cardiac surgery at Cabrini Hospital, Malvern, Victoria, Australia between August 2011 and December 2012. The study protocol was assessed and approved by the Cabrini Institute clinical ethics committee. STROBE criteria were adhered to.

Patients were eligible for inclusion if they were 18 years or older, and able to give informed consent. Patients were recruited consecutively as they arrived in the intensive care unit (ICU) post elective cardiac surgery. Patients with end stage renal disease (ESRD) treated with renal replacement therapy (dialysis or transplantation) were excluded. Additionally those with no available routine pre-operative urinalysis were excluded from the study.

### Study variables

All patients undergoing on pump cardiac surgery had urinalysis for proteinuria and serum urea and creatinine determined within one month prior to surgery. Where multiple determinations of serum creatinine were performed, the highest value was used as the baseline figure. Serum urea and creatinine were subsequently collected immediately upon arrival to ICU, within two hours of surgery completion, daily for 48 h, and prior to discharge. Pre-operative estimated glomerular filtration rate (eGFR) was calculated using the Chronic Kidney Disease Epidemiology Collaboration (CKD-EPI) equation [11]. All blood samples were analysed by the same laboratory.

Urinalysis was performed using Roche Combur 10 Test Strips and proteinuria was graded according to severity ranging from no detectable protein at 0 to a maximum score of 3 as annotated on the urinalysis kit. Subjects were classified as having proteinuria if the test strip score was 1 or higher.

Baseline demographic and covariate data were collected by history and chart review. Intraoperative information collected included type of cardiac surgery, minimum core temperature, total bypass time, cross clamp time and inotropic requirement. Use of peri-operative statins, angiotensin II receptor blockade (ARB), angiotensin converting enzyme inhibitors (ACEI) and antibiotic requirement were noted. Post operative use of inotropes for greater than one hour in the first 24 h, need for renal replacement therapy and in-hospital death were recorded in addition to ICU and hospital length of stay.

### Outcome definitions

Acute Kidney Injury (AKI) was defined and staged according to the Acute Kidney Injury Network (AKIN) criteria. Stage 1: rise in serum creatinine of ≥ 26.4 μmol/L or 1.5 times baseline sCr. Stage 2: increase of ≥ 2–3 fold from baseline sCR; Stage 3: increase of > 3 fold from baseline sCR, a serum creatinine of ≥ 354 μmol/L with an acute increase of at least 44 μmol/L or initiation of RRT, all within 48 h. The percentage change in serum creatinine was then calculated and further divided into 3 categories: increase of ≥ 15% from baseline; decrease of ≥ 15% or within 15% of baseline (control group). This figure was chosen to take into consideration inter-assay variability or small changes that did not reflect true changes in GFR.

### Statistical analysis

Baseline differences between the AKI groups were assessed using t-tests or analysis of variance for continuous variables and the chi-squared test or Fisher’s exact test for categorical variables where appropriate. Logistic regression was used to assess predictors of AKI incorporating the significant pre-operative factors associated with AKI as well as demographic factors including age and gender to create a baseline prediction model. Stepwise manual backward elimination was used beginning with the variable with the highest p-value. Performance of a likelihood ratio test were used to confirm that deleted factors did not contribute to the model. Age and gender were kept in the final model. We then added the immediate postoperative serum creatinine (or eGFR) to the baseline model and compared risk prediction and model discrimination using receiver operating characteristic (ROC) curves. An area of > 0.8 was assessed as good model discrimination. Multicolinearity was checked by assessment of the uncentred variance inflation factors. We considered a finding to be statistically significant if the two-sided *P*-value was < 0.05. All analyses were conducted using Intercooled Stata 12.0 (StataCorp, College Station, Texas, USA).

## Results

We identified 200 consecutive patients who underwent elective cardiac bypass surgery. Of these four were excluded from the study - one patient with ESRD treated with RRT and 3 patients who did not have pre-operative urinalysis leaving 196 patients available for analysis.

Baseline characteristics of the cohort, classified by AKI, are summarised in Table 1. The study population was predominantly male (73.5%) with the median age of enrolled participants being 70.8 years (range 30–91). The mean pre-operative creatinine was 86 μmmol/L (range 40–246; SD 27.0) with a mean eGFR of 75.63 mls/min/1.73 m^2^ (range 21.59–118.8: SD 18.75). 19% of the cohort had diabetes.

**Table 1:** Baseline characteristics of the study population overall and according to the presence or absence of AKI

	Total	No AKI	AKI	p-value
	*N* = 196	*N* = 156	*N* = 40	
Age, median (IQR)	70.8 (63.0, 81.2)	69.7 (61.2, 80.5)	75.2 (66.9, 82.2)	0.035
Male	144	111 (71.2%)	33 (82.5%)	0.15
Operation type				0.59
CABG Only	72	59 (37.8%)	13 (32.5%)	
Valve Only	61	48 (30.8%)	13 (32.5%)	
CABG & Valve	56	42 (26.9%)	14 (35.0%)	
Other	7	7 (4.5%)	0 (0.0%)	
Diabetes mellitus	37	25 (16.0%)	12 (30.0%)	0.044
Ischaemic heart disease	92	70 (44.9%)	22 (55.0%)	0.25
Hypertension	133	100 (64.1%)	33 (82.5%)	0.026
COAD	10	7 (70%)	3 (30%)	0.440
ACEI/ARB use	116	88 (56.4%)	28 (70.0%)	0.12
Pre Op Creatinine (umol/L), median (IQR)	82.0 (71.0,96.0)	80.0 (69.5, 93.0)	90.0 (77.5, 119.5)	0.003
Pre Op eGFR (ml/min/1.73 m^2^), median (IQR)	80.1 (64.3, 88.9)	82.0 (66.5, 89.6)	69.4 (49.2, 81.5)	0.002
Pre Op proteinuria	35	23 (14.7%)	12 (30%)	0.025
Pre Op CKD stage^a^				0.013
Stage 1	41 (21%)	37 (23%)	4 (10%)	
Stage 2	117 (60%)	96 (62%)	21 (53%)	
Stage 3a	23 (12%)	15 (10%)	8 (20%)	
Stage 3b	13 (7%)	7 (4%)	6 (15%)	
Stage 4	2 (1%)	1 (1%)	1 (3%)	
Cardiopulmonary bypass time (mins), median (IQR)	96.1 (77.1, 129.9)	96.7 (77.1, 129.9)	90.7 (76.6, 131.5)	0.94
Cross clamp time (mins), median (IQR)	77 (60–109)	79.3 (61,107)	75.63 (60,113)	0.94
Inotrope use	112	83 (53.2%)	29 (72.5%)	0.028
Total length of stay (days), median (IQR)	10.0 (8.0,13.0)	9.0 (8.0, 12.0)	11.5 (9.0, 16.5)	0.002
ICU length of stay (days), median (IQR)	1.0 (1.0,2.0)	1.0 (1.0, 2.0)	2.0 (2.0, 3.5)	<0.001

No subject had stage 5 CKD

*AKI* Acute kidney injury, *IQR* interquartile range, *CABG* coronary artery bypass surgery, *DM* diabetes mellitus, *IHD* ischaemic heart disease, *COAD* chronic obstructive airways disease, *ACE* angiotensin converting enzyme inhibitor, *ARB* angiotensin receptor blockade, *eGFR* estimated glomerular filtration rate, *ICU* intensive care unit

^a^CKD Stage defined using eGFR only

### Clinical outcomes

Of the 196 patients enrolled in the study, 40 (20.4%) developed AKI. The majority of AKI was stage 1 (35/40 subjects [87.5%]), with similar proportions classified as stage 2 (3/40 subjects [7.5%]) and stage 3 (2/40 subjects [5%]). Two patients required renal replacement therapy and there were two deaths, both occurred in subjects with stage 3 AKI. Subjects with AKI tended to be older (median age 75.2 vs 69.7 years; *p* = 0.023), have more diabetes (30% vs 16% *p* = 0.04) and have poorer pre-operative kidney function (eGFR 69 vs 82 ml/min/1.73 m^2^, *p* = 0.002). CKD stages 3 and 4 were also more common in patients with AKI (38% vs 16%, *p* = 0.013). Patients with AKI required a longer stay in ICU with a mean duration of 2.8 days versus 1.6 days for those patients who did not develop AKI (*p* < 0.001). Total length of stay was also longer in patients with AKI (mean duration 11.1 vs 15 days *p* = 0.002). At hospital discharge, the eGFR was significantly lower in the AKI group compared to the no AKI group (46 versus 84 ml/min/1.73 m^2^ *p* < 0.001) with corresponding mean serum creatinine levels of 115 and 79 μmol/L respectively (*p* < 0.001).

### Baseline predictors of AKI

**Fig. 1:**
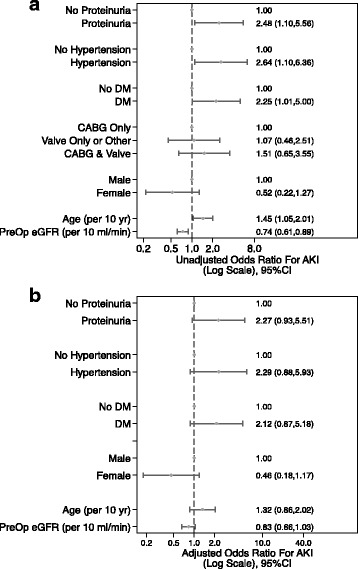
**a** Pre-operative factors associated with an increased odds of acute kidney injury (unadjusted analysis). **b** Risk factors associated with an increased odds of acute kidney injury – baseline pre-operative model (adjusted analysis)

**Fig. 2:**
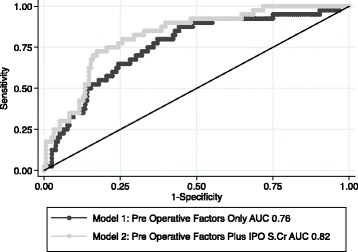
Receiver operating characteristic (ROC) curves comparing baseline model 1 (baseline pre-operative model) to model 2 (baseline pre-operative model with addition of change in immediate post-operative serum creatinine)

On univariate logistic regression analysis (Fig. 1a), pre-operative factors significantly associated with an increased likelihood of AKI were hypertension (OR 2.64, 95% CI 1.10–6.36; *p* = 0.02), diabetes mellitus (OR 2.25, 95% CI 1.01–5.0; *p* = 0.04), and proteinuria (OR 2.48, CI 1.1–5.56; *p* = 0.02). Higher pre-operative eGFR was protective (OR 0.74 per 10/ml/min/1.73 m^2^, CI 0.61–0.89; *p* = 0.002). A baseline multivariate model was constructed incorporating the significant pre-operative factors associated with AKI as well as demographic factors including age and gender. This initial model included age, gender, baseline kidney function as assessed by eGFR, pre-operative proteinuria, hypertension, diabetes and type of cardiac surgery. Following manual stepwise removal based on likelihood ratio tests, the final model included eGFR, pre-operative proteinuria, hypertension, and diabetes (Fig. 1b). Model discrimination for this baseline model was moderate at best with an area under the ROC curve of 0.76 (95% CI 0.68–0.84) (Fig. 2).

### Early post-operative creatinine predicting AKI

On univariate analysis, compared to a change of <15% in the early post operative serum creatinine, a rise of more than 15% over baseline was associated with an increased likelihood of developing AKI (OR = 3.44; 95% CI 0.72–16.55; *p* = 0.122) however this was not statistically significant. A fall of >15% from baseline in the early post-operative serum creatinine was protective for the development of AKI (OR = 0.34; 95% CI 0.16–0.73; *p* = 0.006). On its own, the change in early post-operative serum creatinine was poorly predictive for AKI (ROC AUC 0.66, 95% CI 0.57–0.74).

**Fig. 3:**
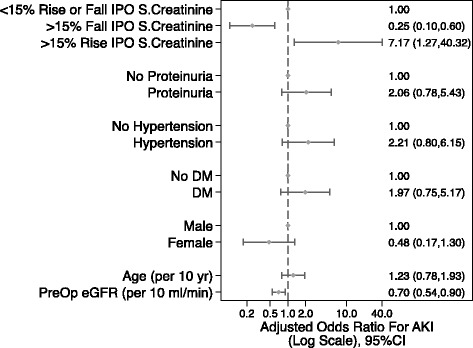
Baseline pre-operative model with addition of change in immediate post-operative serum creatinine

We then tested the predictive value of adding the percentage change in early postoperative serum creatinine to the developed baseline pre-operative AKI model. After adjustment of pre-operative AKI risk factors, a 15% increase in the immediate post operative serum creatinine was significantly associated with a 7-fold increase in the likelihood of AKI (OR 7.17; 95% CI 1.27–40.32; *p* = 0.025) (Fig. 3). Likewise a >15% fall in the serum creatinine was protective (OR 0.25; 95% CI 0.10–0.60; *p* = 0.002). The addition of immediate post-operative serum creatinine to the baseline model improved the predictive ability with an area under the curve ROC curve to 0.82 (95% CI 0.75–0.89) indicating improved model discrimination although the difference in the ROC AUC did not reach statistical significance (*p* = 0.07 vs base model) (Fig. 2).

## Discussion

In this prospective study of AKI post elective cardiac surgery we have demonstrated that AKI is common (20% incidence in our study) and is associated with an increase in both hospital and ICU length of stay. A simple model of pre-operative patient characteristics and AKI risk factors combined with a >15% rise or fall in the immediate post-operative serum creatinine was significantly predictive of the development of AKI, thus enabling the identification of patients at risk of AKI early in their hospital stay without the need for specialized tests or assays. The identification of patients with AKI is important as in addition to increased short-term morbidity and mortality, AKI is associated with a doubling of mortality extending to 10 years, even when renal function returns to normal [12]. Additionally, subjects with AKI are at risk of developing CKD in the long term [3, 13].

While few interventions have been definitively proven to improve outcomes in AKI, early nephrology referral has been shown to be beneficial in a number of studies. In a 2002 study of ICU patients with AKI, delayed referral to specialist nephrology services was associated with increased length of stay and higher mortality rates [5]. In a pilot study of 178 patients, nephrology involvement within 18 h of the development of AKI, with diagnostic and therapeutic interventions, resulted in improved outcomes [6]. Similarly in a cohort of Brazilian patients admitted to ICU with AKI, delayed consultation was associated with higher mortality [7]. Despite this data, referral rates to nephrologists are low, and were approximately 50% in the previous studies. Disturbingly, less that 10% of 4000 patients who developed AKI in hospital were referred for outpatient nephrology consultation [14]. Early diagnosis of AKI is important; firstly, it will help identify patients for nephrology referral and secondly, it will allow for timely interventions, which could improve outcomes.

In the past decade there has been increasing interest in incorporating urine and serum biomarkers of AKI into clinical practice. It is anticipated that these biomarkers will enable earlier diagnosis of AKI and facilitate prognostication [15]. In a prospective study of over 1200 adults undergoing cardiac surgery [8] patients within the highest quintile of urine IL-18 production demonstrated a near 7-fold increase in AKI, while AURoC values improved from a baseline of 0.69 to 0.76. Similarly in patients with elevated serum NGAL, rates of AKI were 5-fold higher with an AURoC of 0.76. These results suggested that measurement of urine IL-18 and NGAL within 6 h after surgery could help predict patients likely to develop AKI. However, further studies involving these biomarkers and others have produced conflicting results and as noted in a recent in-depth review, a significant benefit over clinical parameters has not been proven [16]. Furthermore, these tests are expensive and not readily accessible to most practitioners; they therefore do not form part of routine clinical practice.

Despite its relative non-specificity, sCr remains the gold standard for defining AKI. Its importance in predicting outcomes after cardiac surgery is well recognized in both adult and paediatric populations. Zappitelli et al [17] published a retrospective study of 390 children undergoing cardiac surgery. They too found that a percentage serum creatinine rise on day 1 post-operatively predicted AKI at 48 h. In a large (>4000 patients) prospective cohort study, over half of the patients developed a drop in sCr after surgery; the mortality rate in these patients was 2.6%. Conversely in patients whose creatinine rose, the mortality rate was 8.9% [18]. The drop in sCr that occurs in most patients is presumably a normal physiological response; conversely, a rise in sCr flags the patient as being at high risk of complications. Ho et al introduced early sCr, measured within 6 h after surgery, into their predictive equation and found that AURoC improved from 0.69 to 0.78. We investigated the utility of sCr measured within 2 h of the completion of cardiac surgery. We found that changes in sCr (and eGFR) accurately predicted the subsequent development of AKI. Furthermore AURoC increased from a baseline value of 0.76 to 0.82. The predictive ability of our baseline model was comparable to that reported by Parikh and colleagues [8]. Inclusion of early sCr into our model matched and even marginally exceeded their model after inclusion of urinary Il-18 and serum NGAL.

The main strengths of our study are a moderately sized patient cohort with a prospective trial design, and use of a simple and readily available test. The limitations are that patients were recruited from a single center, AKI was assessed at 48 h only and there was a low rate of stage 3 AKI.

## Conclusions

In summary, we found that a rise of >15% in sCr over baseline, taken within 2 hours of arrival to ICU, equated to an odds ratio of 7 for the development of AKI. This is important as sCr is an inexpensive, readily available test and measurements of early sCr could easily be incorporated into clinical practice, even in resource poor settings. We suggest that patients who demonstrate an increase in early sCr should be referred to a nephrologist in a timely manner. This would enable early interventions and potentially improve outcomes.

## Abbreviations

AKI: Acute Kidney Injury
AKIN: Acute Kidney Injury Network
CKD: Chronic kidney disease
eGFR: Estimated glomerular filtration rate
ESRD: End stage renal disease
ICU: Intensive care unit
IPOsCr: Immediate post operative serum creatinine
NGAL: Neutrophil gelatinase-associated lipocalin
sCR: Serum creatinine
